# From Virtual Molecules to Clinical Trials: How AI Is Reshaping Preclinical Drug Discovery

**DOI:** 10.2196/101366

**Published:** 2026-05-29

**Authors:** Benedette Cuffari

**Keywords:** artificial intelligence in drug discovery, machine learning in drug design, generative chemistry, de novo drug design, computational drug discovery

## Abstract

Pharmaceutical development is typically a long and arduous process, but advances in artificial intelligence may help streamline each stage of this pipeline. In this *News and Perspectives* article, JMIR Correspondent Benedette Cuffari reports on recent innovations and their potential implications for drug discovery.

**Key Takeaways:**

- Artificial intelligence is transforming preclinical drug discovery by enabling more efficient identification, design, and optimization of drug candidates through machine learning, deep learning, and generative modeling approaches.
- Virtual screening, molecular docking, and de novo design methods are increasingly integrated into drug discovery pipelines to improve prediction of drug-target interactions and accelerate lead optimization.

From “bench to bedside,” drug discovery and development is a highly complex process that typically takes 10 to 15 years or more before a new molecule reaches the market. Drug development typically begins with the identification and validation of a biological target, such as a disease-associated receptor, enzyme, or signaling protein. Thereafter, candidate molecules are subjected to in vitro high-throughput screens (HTS) to optimize drug activity, target affinity, and toxicity profiles before a compound undergoes extensive preclinical and clinical testing. Collectively, the development of a single drug molecule is valued at an average of US $2.6 billion, with these high costs attributed to the sequential nature of each stage.

Recent advancements in artificial intelligence (AI) technologies are enabling faster, data-driven identification and optimization of drug candidates, with the potential to reduce both development time and costs. These advancements are transforming every stage of drug discovery, from target identification to molecule design and optimization.

## Machine Learning in Drug Discovery

Machine learning (ML) is a subset of AI that enables systems to learn labeled or unlabeled data from chemical libraries, biological assays, and clinical trials; predict outcomes; and generate actionable insights. ML methods were originally introduced into the drug discovery process in the 1980s and 1990s, during which random forests, support vector machines (SVMs), and other ML algorithms were utilized to model layered relationships between molecular structure and biological activity based on learned datasets.

By the early 2000s, deep learning (DL) algorithms capable of processing vast amounts of data enhanced the ability of these systems to predict structural and molecular properties. Specifically, biological and chemical data acquired from HTS and other multiparameter analyses are analyzed to model and predict drug-target interactions. Modern DL tools involved in drug discovery include graph neural networks—a powerful molecular modeling system that predicts solubility and protein-ligand interactions—whereas transformer architectures model long-range dependencies and contextual relationships during target/drug prioritization.

About 90% of drug candidates fail during phase I, II, or III clinical trials, with unsuccessful outcomes primarily attributed to a lack of clinical efficacy, as well as unmanageable toxicity, suboptimal drug-like properties, and poor strategic planning. To improve the safety of new medications, AI models, like quantitative structure-activity relationship (QSAR) modeling and DL, have been developed to assess a wide range of absorption, distribution, metabolism, excretion, and toxicity features. ChemMORT, for example, is a recent DL-based analytical platform that relies on a simplified molecular input line entry system (SMILES) encoder, descriptive decoder, and molecular optimizer to better predict how drug candidates will behave within biological systems.

## Why Drug Development Remains Expensive

Despite recent developments in computing power, pharmaceutical innovation has not become proportionally faster or cheaper. This contrast is often described as Eroom’s Law—the observation that drug development becomes more expensive over time, even as digital technologies rapidly advance.

Mark Gerstein, PhD—professor of biomedical informatics, molecular biophysics, and computer science at Yale University—said that this paradox helps explain why enthusiasm around AI should be tempered with realism. “There is a lot of excitement with AI in drug discovery,” Gerstein said, “but the reality is that we have not been as successful at discovering drugs as we would have hoped.”

Computers continue to improve under Moore’s Law—the approximate doubling of transistors on microchips every 2 years—and the cost of genome sequencing has fallen dramatically. Nevertheless, these efficiencies have not eliminated the biological and clinical challenges that drive failure rates and development costs.

**Figure:**
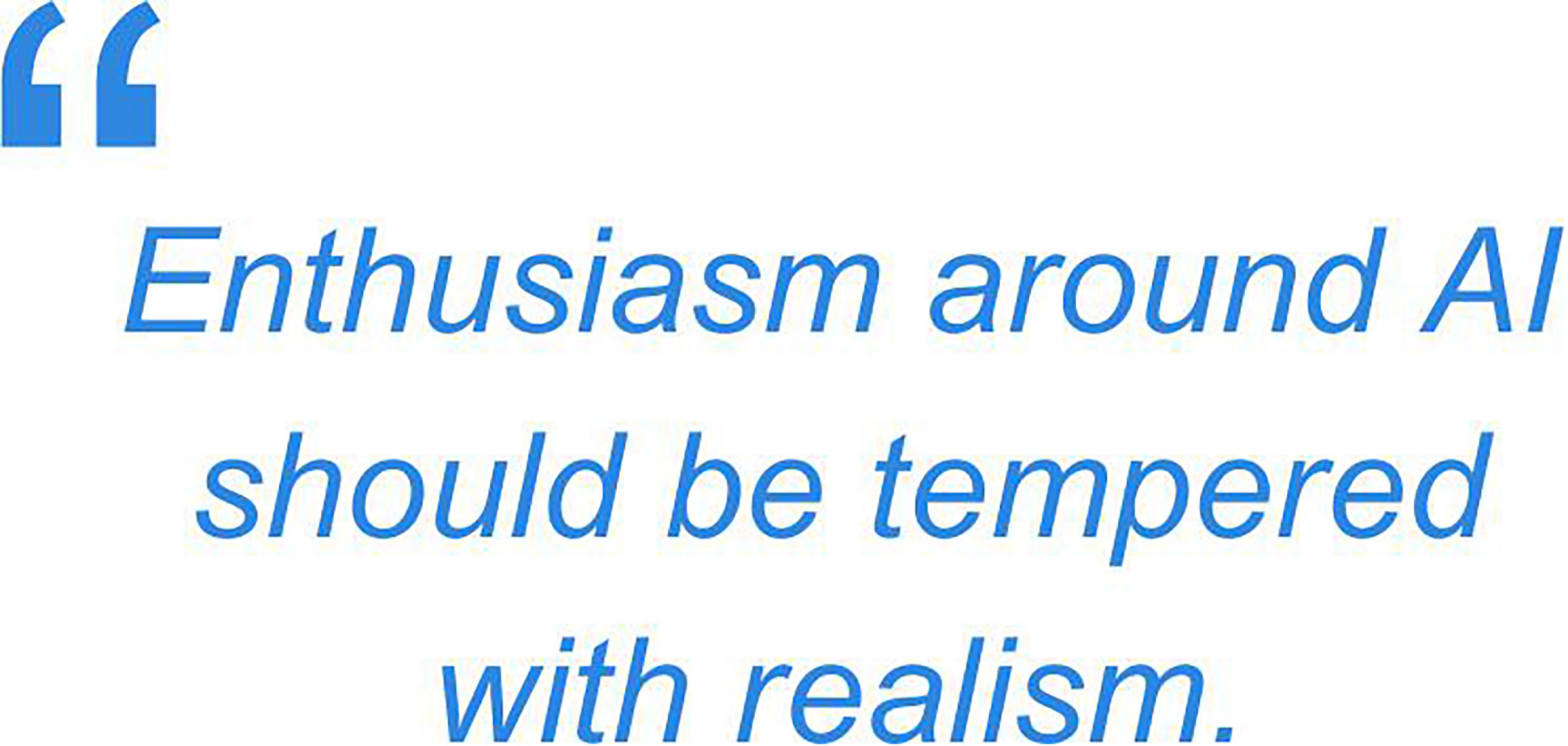


## Structure-Based AI Design

Generative chemistry, otherwise known as “drug design,” involves the creation of novel molecular compounds intended to be stable, valid, and unique drugs with pharmaceutical potential. Using both ML and DL algorithms, virtual screening is frequently involved in the early stages of drug development to learn the structures and properties of enormous compound libraries and predict their potential interactions with a specified drug target.

Structure-based virtual screening (SBVS), for example, leverages molecular docking to identify drug candidates based on their predicted interactions with a target protein. ML algorithms, like SVMs and random forest models, predict ligand binding within the protein’s active site, allowing medicinal chemists to rationally modify functional groups to improve binding affinity.

Gerstein also points to equivariant neural networks as a promising advance in AI-driven drug discovery. Since molecules obey physical laws and can rotate, stretch, or change their conformation, predictive models must account for these transformations. Equivariant architectures are designed to preserve these relationships, making them especially valuable for molecular modeling and binding prediction.

Compared with ML-based methods used to create drug-like candidates based on existing chemical libraries, de novo design can be applied to generate new compounds with specific potency and selectivity properties. To this end, generative models, like variational autoencoders and generative adversarial networks (GANs), can be trained on a dataset of known molecules to learn their structural properties and generate molecules with similar properties. Reinforcement learning algorithms are also used during de novo molecule design to optimize the chemical structure of a new drug based on its pharmacological properties, with several hybrid approaches emerging that integrate different aspects of each model.

## AI-Driven Drug Discovery Platforms and Clinical Translation Partnerships

Exscientia, Insilico Medicine, Recursion, BenevolentAI, and Schrödinger are five leading AI-derived drug discovery companies that have successfully advanced novel small-molecule drug candidates into the clinic. Both rentosertib (ISM001-055)—a pulmonary fibrosis candidate developed by Insilico Medicine—and DSP-1181—a serotonin (5-HT1A) receptor agonist developed by Exscientia—were designed by using AI-driven platforms before advancing to early clinical testing.

BenevolentAI and AstraZeneca joined forces in 2019 to identify novel targets in chronic kidney disease and idiopathic pulmonary fibrosis by using AI-driven knowledge graphs. Collectively, these partnerships highlight that drug discovery is shifting from isolated computational innovation toward integrated pipelines in which AI platforms and pharmaceutical companies drive target identification, molecule design, and clinical translation.

## Challenges and Limitations

The integration of AI techniques into drug design has several advantages over traditional discovery methods, including reduced time and costs, as well as better accuracy in predicting the safety and efficacy of new drugs. Nevertheless, the lack of diverse, representative, and high-quality datasets may introduce bias that leads to inaccurate predictions. In fact, many available chemical and biological datasets are incomplete or are acquired from heterogeneous experimental conditions, thereby reducing model generalizability and applicability to new chemical structures.

Trust is also imperative, underscoring the importance of transparency through open-source models, shared datasets, and explainable frameworks. However, AI does not eliminate downstream failures in clinical development. Gerstein cautions that once a promising molecule is identified, substantial challenges remain in optimizing delivery, systemic effects, and safety. Biological complexity, patient heterogeneity, and unforeseen toxicity mechanisms perpetuate high attrition rates, highlighting the need for AI systems that integrate more robust biological clinical context.

